# Characterization of COVID-19 vaccine clinical trial discussions on the social question-and-answer site Quora

**DOI:** 10.1186/s13063-023-07837-5

**Published:** 2023-12-05

**Authors:** Qing Xu, Tiana J. McMann, Jiawei Li, Christine Wenzel, Tim K. Mackey

**Affiliations:** 1 Global Health Policy and Data Institute, San Diego, CA USA; 2 S-3 Research, LLC, San Diego, CA USA; 3 Global Health Program, Department of Anthropology, University of California, San Diego, CA USA

**Keywords:** COVID-19, Vaccine, Clinical trial, Quora, Social listening, Health equity, Retrospective analysis, Data mining

## Abstract

**Introduction:**

Throughout the COVID-19 pandemic, underserved populations, such as racial and ethnic minorities, were disproportionately impacted by illness, hospitalization, and death. Equity in clinical trials means that the participants in clinical trials represent the people who are most likely to have the health condition and need the treatment that the trial is testing. Infodemiology approaches examining user conversations on social media platforms have the potential to elucidate specific barriers and challenges related to clinical trial participation, including among racial and ethnic minority populations.

**Materials and methods:**

The study retrospectively collected and analyzed user question and answer posts from Quora in October 2021 using an inductive content coding approach. We also examined user’s publicly available profile metadata to identify racial and ethnic minority users to capture their experiences, attitudes, topics, and discussions of barriers to COVID-19 vaccine trials.

**Results:**

A total of 1073 questions and 7479 answers were collected. A total of 763 questions and 2548 answers were identified as related to COVID-19 vaccine clinical trials. The majority of these online interactions focused on asking questions and sharing knowledge and opinions about COVID-19 vaccine trials, including major topics related to: (a) interpreting whether clinical trial results could be trusted; (b) questions about vaccine efficacy and safety; (c) understanding trial design, regulatory considerations, and vaccine platforms; and (d) questions about trial enrollment, length, and adequate representation. Additionally, four major barriers discussed included: (i) disagreement from users regarding whether clinical trials require representation from different racial and ethnic minorities; (ii) concerns regarding the safety of trials when participating; (iii) lack of knowledge on how to participate in a trial; and (iv) questions of whether participants could withdraw from a trial to access an approved COVID-19 vaccine.

**Conclusions:**

Our study found active user discussions related to COVID-19 vaccine clinical trials on Quora, including those specific to minority health topics and those posted by a smaller group of self-identified racial and ethnic minority online users. Results from this study can help identify barriers to participation among the general public and underrepresented groups while also supporting the design of future outreach strategies to help with recruitment and inclusive trial participation.

**Supplementary Information:**

The online version contains supplementary material available at 10.1186/s13063-023-07837-5.

## Introduction

The COVID-19 pandemic is an unprecedented public health emergency that has led to over 760 million cases globally as of mid-2023, with the United States leading all countries in the number of confirmed cases and deaths [1]. However, the disease burden of COVID-19 has been unevenly distributed, with racial and ethnic minority populations suffering a disproportionate COVID-19 burden across rates of infection, hospitalization and course of treatment, and prognosis, including for morbidity and mortality [2, 3]. In the United States, cases, hospitalizations, and death rate ratios are higher among American Indian or Alaskan Native, Asian, Black or African American, and Hispanic or Latino populations when compared with Non-Hispanic White populations [4]. Additionally, these same populations are more likely to have an intensive care unit admission when compared to Non-Hispanic White populations, underlying the severity of COVID-19-associated outcomes, the importance of prevention, and the impact of social determinants of health and health equity factors [5].

The pandemic has also clearly demonstrated the importance of investment in and rapid development of vaccines and other medical countermeasures to slow the spread and mitigate the severe economic, social, and health consequences of this novel virus [6]. However, disparities in vaccine uptake have been observed among certain U.S. populations who have remained at increased risk for COVID-19 and associated complications, despite efforts to improve vaccine availability and accessibility [1, 7]. Disparities in vaccine uptake and high rates of vaccine hesitancy further demonstrate the importance of racial and ethnic minority engagement with the clinical trials process in order to establish trust in the vaccine and therapeutic development among these communities, yet these groups remain historically underrepresented in clinical trials, including for cardiology, mental health, diabetes trials, and also COVID-19 trials [8, 9].

To advance efforts and understanding of diverse patient perspectives and attitudes, approaches using infodemiology (i.e., studying the determinants and distribution of information in an electronic medium to inform public health) can better identify and characterize lived experiences and attitudes towards COVID-19 clinical trials and vaccination, particularly when analyzing publicly available social media data [10–12]. Hence, the aim of this study was to leverage a unique and understudied social media question-and-answer platform, Quora, to identify specific experiences and attitudes regarding challenges related to COVID-19 vaccine clinical research participation, including those topics related to health equity and racial and ethnic minority populations. Based on these results, we hope to provide additional insights into the unique challenges faced by underrepresented communities in participating in COVID-19 and other clinical trials.

## Materials and methods

This study was conducted in two phases: data collection and data analysis. We collected and analyzed publicly available data from Quora (www.quora.com), a social question-and-answer online community platform, where users actively engage in discussions about COVID-19, vaccines, clinical trials, and other health-related topics. As of 2020, it was reported that Quora had 300 million users per month. We also attempted to determine if user discussions were related to specific racial and ethnic minority and/or health equity topics or if users self-reported as a member of a racial or ethnic minority group based on their publicly available information.

### Ethical considerations

As this study only analyzed secondary publicly available data and does not report any individually identifiable information on users, it was deemed exempt by WCG IRB.

### Data collection

Quora is a popular publicly available Q&A platform where users can post, follow, share, answer, and comment on questions and upvote answers. Each Quora user has a profile in which users can voluntarily self-report their demographic information, educational background, working experience, and the platform also reports their number of followers and followees. To collect posts potentially related with COVID-19 vaccine clinical trial topics, we first generated two lists of keywords associated with the COVID-19 vaccine and clinical trials by manually searching posts on the Quora platform. We used “COVID vaccine” and “clinical trial” as our baseline general terms and searched these two keywords on Quora’s platform search query function, which enabled us to collect additional keywords associated with the COVID-19 virus, COVID-19 vaccine, and clinical trial experiences as observed from the first 30 returned results from each initial search term used. This enabled us to generate a more comprehensive list of COVID-19 vaccine and clinical trial- keywords used in conversations on Quora (see Table 1 for the full list of study keywords) that were then used for additional structured searches and data collection.

**Table 1:** Selected “COVID-19 vaccine” and “clinical trial” related key words

	Additional keywords
COVD-19 vaccine related keywords	COVID-19 vaccine; COVID 19 vaccine; SARS-CoV2 vaccine; coronavirus disease 2019 vaccine; mRNA-1273; Pfizer; BioNTech; BNT162; Moderna; J&J; Johnson & Johnson
Clinical trial related keywords	Clinical trial; trial

The Quora platform does not currently offer publicly available datasets or an official application programming interface. In order to automate the collection of user-generated data from Quora, we developed an automated data mining script using the programming language Python to collect publicly available questions and answers from Quora based on keyword searches, as well as the profile information related to users returned in results (including aforementioned publicly available metadata of a user account). Quora questions containing both COVID-19 vaccine-related keywords and clinical trial-related keywords and the user-generated answers associated with these questions were collected and analyzed for inclusion in this study. Data were retrospectively collected in October 2021, including the textual content of the question, the number of answers to the question, the textual content of each underlying answer, user profile information of users who answered the original question, and upvote and downvote numbers.

### Data analysis

#### Qualitative content analysis

Our content analysis focused on detecting themes related to knowledge, experiences, and barriers related to COVID-19 vaccine clinical trials, specifically, attitudes to participating or not wanting to participate in a COVID-19 vaccine clinical trial. To classify the content of collected data, two coders (QX and TJM) first independently used a binary coding approach to filter collected Quora questions for relevance to the topic of COVID-19 vaccine clinical trials and excluded Quora questions that did not meet the objective of this study (e.g., questions related to vaccines for other diseases, questions related to other non-vaccine COVID-19 trials, etc.). We then filtered all answers confirmed as related to COVID-19 vaccine clinical trial-related questions and analyzed their answers by combining the original question and an answer as one answer post unit to represent the complete answer information. We then used a general inductive coding approach to conduct in-depth qualitative coding of the textual data of filtered answers. First, all answers were reviewed by the first author (QX), and notes were taken on general themes of posts from which the initial code list was created. Next, formal coding of text data was conducted with codes refined, and subcodes developed. Finally, the second author (TJM) reviewed the final coded dataset, and the first and second authors reconciled differences in code definitions and application. First (QX) and second (TJM) authors coded all posts independently and achieved high intercoder reliability for Quora question classification (Cohen κ = 91.56). Based on the content of collected answers, all detected themes were classified into two major themes: 1) COVID-19 clinical trial participation; and 2) COVID-19 clinical trial design, process, and results. Descriptive statistics of data characteristics and distribution of the volume of topics coded, including from a timeline perspective, were also carried out.

#### Topic interaction analysis

To further analyze the popularity of different topics related to COVID-19 vaccine clinical trials, we also examined the volume of users’ interaction behavior for relevant questions and answers. The popularity of questions was analyzed by using the number of total answers, and the popularity of answers was analyzed by using the number of upvoting and downvoting and the number of sharing of the answer.

#### User metadata analysis

To identify the specific challenges and barriers related to COVID-19 vaccine clinical trial participation, specific to racial and ethnic minority populations, we examined publicly available Quora profile metadata of users with answers relevant to the study aims. This study included four racial minority groups: Blacks or African Americans, American Indians and Alaska Natives, Asians, Native Hawaiian or other Pacific Islanders, and the ethnic minority group: Hispanics or Latinos. We classified users’ minority status by only using publicly available self-reported profile information of racial and ethnic status from textual data. In order to further filter the data, we first identified users’ residency status based on the user’s self-reported current living city/country. Though not included in this analysis, user’s educational experience and working experience information from the user’s Quora profile or “Credentials & Highlights” section was also collected. We then filtered for all users residing in the United States and further categorized them into the four racial minority groups and one ethnic minority group. Though some Quora profiles included images or profile pictures, these were not used for the purpose of racial and minority classification due to low volume of the number of pictures for users with answers, common use of pictures that were clearly not the user (e.g., logo, unrelated character, animal/pet, etc.), and the general ambiguity of user profile pictures that may not clearly represent a specific racial or ethnic minority classification (e.g., mixed race). Data were collected for purposes of aggregation, and no results contained in this study includes individually identifiable information or makes any representation of the accuracy of a claimed minority or ethnic classification of a user.

## Results

### Overview

Our data collection approach yielded 1073 questions and 7479 answers from Quora. Based on all collected data, both the oldest question and the oldest answer were posted on May 10, 2019 (before the pandemic and which was excluded from analysis), and the most recent question and answer were published on October 7, 2021, and October 20, 2021 (near the date of data collection commencement). After applying our binary coding approach, we identified 508 (47.34%) questions related to COVID-19 vaccine clinical trials. In addition, based on our manual annotation and content coding, we identified 3105 answers from selected relevant questions, of which 2542 (33.99%) answers were identified as related to COVID-19 vaccine clinical trial-related topics, which comprised responses from a total of 1154 unique Quora user accounts. From these relevant posts, the timeline of the Quora questions and answers that included readable timestamps had the earliest question dated May 1, 2020, and the latest question dated October 7, 2021. Corresponding to these questions, the oldest answer was posted on January 10, 2020, and the most recent response was posted on October 17, 2021.

### Content analysis

According to our qualitative analysis and inductive coding approach, 28 topics were derived under two major parent domains and seven subcodes (refer to Table 2 for a complete breakdown of the stratification of these codes and anonymized and paraphrased examples from Quora). According to our inductive coding, all detected topics could be classified into two major domains: COVID-19 vaccine clinical trial participation (491/2542, 19.32%); and COVID-19 vaccine clinical trial design, process, and results (2051/2542, 80.68%).

**Table 2:** Code list and Identified Topic Themes

Code Name	Code number	Description	Number of Post	Upvote number
**(A) Trial Participation**				
**(A-1) People Enrolled**	(A-1-a)	Comments concerning the safety of the trial	44	46
	(A-1-b)	Cost vs. benefits of staying in the trial after a vaccine has already been approved	9	4
	(A-1-c)	Sharing the reasons for volunteering for a COVID-19 vaccine clinical trial	27	140
	(A-1-d)	Sharing or asking participants about experiences including side effects and transparency of the trial data	61	117
	(A-1-e)	Sharing COVID-19 vaccine clinical trial information (knowledge – educate other users about knowledge related to a clinical trial)	61	276
	(A-1-f)	Discussing whether participants will be able to get vaccinated when a vaccine is approved	36	67
**(A-2) People Who Want to Participate**	(A-2-a)	Seeking or sharing volunteer opportunities (location, website)	27	106
	(A-2-b)	Discussing the willingness or unwillingness of participating in a clinical trial and reasons (pediatric opportunities, not willing to participate)	226	422
**(B) Clinical Trial Design/ Process/ Result**				
**(B-1) Length of Trial**	(B-1-a)	Discussing the time period of one or more clinical trials being too long	12	12
	(B-1-b)	Discussing the period of one or more clinical trial not being long enough	6	12
**(B-2) Eligibility of Trial**	(B-2-a)	Discussing topics about trial participant representation (self-selection bias, how they are selected, number of participants)	59	123
	(B-2-b)	Discussing health condition representation (comorbid conditions, pregnancy)	14	21
	(B-2-c)	Discussing different age representation in COVID-19 vaccine clinical trials (kids/elderly can’t participate)	42	253
	(B-2-d)	Discussing the racial representation of COVID-19 vaccine clinical trials	11	283
**(B-3) Trial Mechanism**	(B-3-a)	Discussing about how efficacy is proven based on the design of the trial (e.g., participants not exposed to virus in trial, how often participants get tested, how to test long-term immunity)	163	445
	(B-3-b)	Discussing the mechanism of developing the COVID-19 vaccine (Pros and cons of mRNA vaccine, the use of electroporation, why use of placebo)	151	694
	(B-3-c)	Discussing topics about COVID-19 emergency use authorization on the design of the trial (fast-track)	83	274
	(B-3-d)	Discussing trial regulations/processes (e.g., double blind, triple blind, process for pausing trial, regulatory agency)	175	770
**(B-4) Trial Stage**	(B-4-a)	Discussing current trial phase (e.g., pause about COVID-19 vaccine trial)	537	1514
**(B-5) Result of Trial**	(B-5-a)	Discussing the general safety of an approved vaccine (if the result of trial can be trust, if the vaccine is safe for teens/kids)	247	25,782
	(B-5-b)	Discussing clinical trials in other countries (e.g., can results of other countries be trusted -Russia, China, India)	189	1909
	(B-5-c)	Discussing topics about the efficacy in trial results	97	264
	(B-5-d)	Discussing topics regarding whether regulations have been followed at trial completion	160	665
	(B-5-e)	Sharing/ discussing about purported vaccine-related safety issues and long-term effects	39	127
	(B-5-f)	Discussing the necessity of vaccines, refusal of vaccines, vaccine hesitancy, alternative therapy	6	8
	(B-5-g)	Discussing or comparing trial results among all different companies	55	492
	(B-5-h)	Discussion of topics related to vaccine manufacturer	5	9

**Fig. 1:**
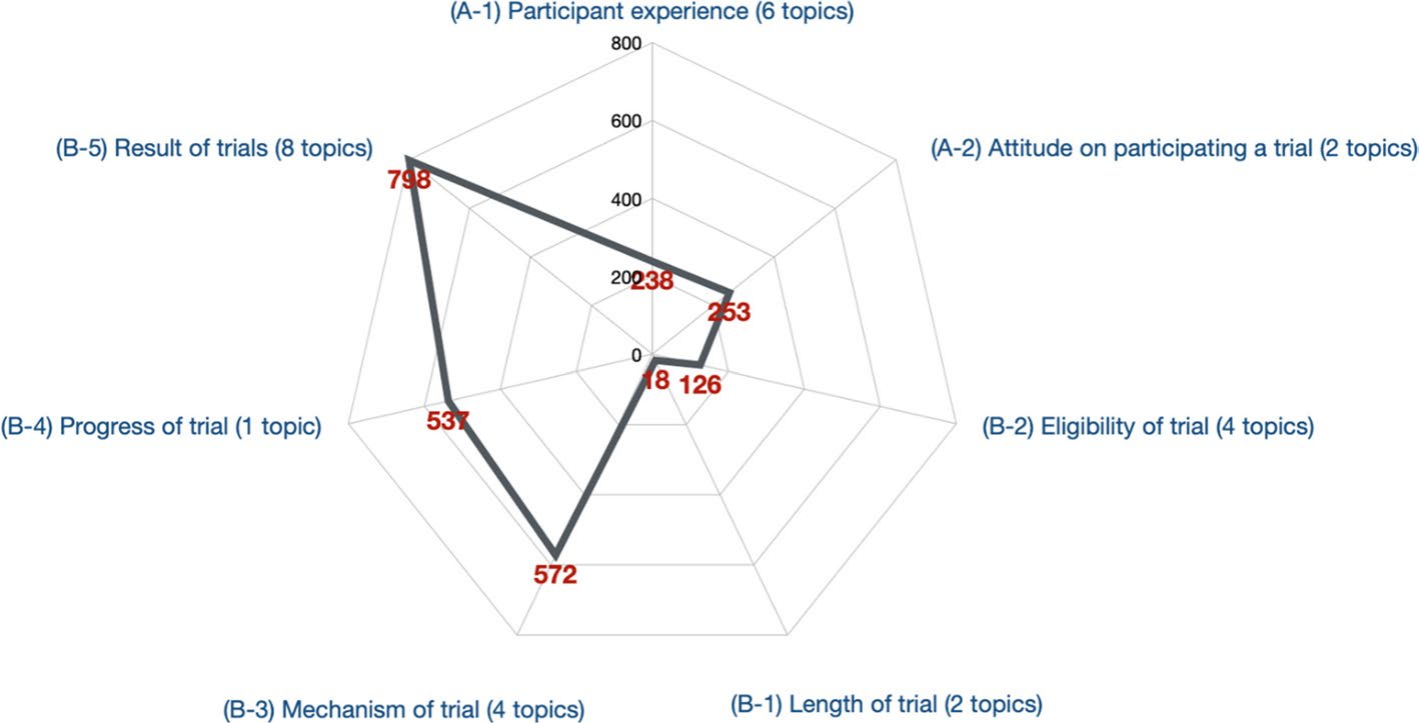
Content Breakdown into Sub-topics

According to the answers identified from the trial participation parent topic (parent code A), which focused on discussions related to participation in COVID-19 vaccine clinical trials, we were able to identify user conversations focusing on sharing specific concerns about trials (A-1-a, A-1-b), trial experiences (A-1-d), trial knowledge (A-1-e, A-1-f), and the reasons (A-1-c) for participating in a COVID-19 vaccine clinical trial as discussed by actual trial participants (*n* = 238) (see Fig. 1 for content classification breakdown). Among these topics, sharing the experience of participating, including topics related to side effects (A-1-d, *n* = 61) and sharing trial knowledge topics (A-1-3, n = 61), had the highest volume of posts, followed by the topic concerning safety of COVID-19 vaccine clinical trials (A-1-a, *n* = 44). We also observed specific concerns from trial participants about trial participation and subsequent vaccine access eligibility. Specifically, users questioned whether if other vaccines were approved during their trial participation, they would be able to get vaccinated with a subsequently approved vaccine or whether they would need to withdraw from trial participation (A-1-b, *n* = 9; A-1-f, *n* = 36). There were also 27 posts that shared the specific reason why a user participated in a clinical trial (A-1-c) including: having watched a family member die from COVID-19 and wanting to prevent the same from happening to others, feeling proud of trial participation based on the belief that they were helping others, and wanting to get a quick and free vaccination. Among this trial participation parent topic, we were also able to detect discussion focusing on sharing trial recruitment information (A-2-a, *n* = 27) and discussion related to willingness and unwillingness to participate in a COVID-19 vaccine clinical trial from those who did not actually participate (A-2-b, *n* = 226). For example, a group of people reported they would rather be infected with COVID-19 virus than get a vaccine, so they would not participate in a clinical trial.

In the clinical trial design, process, and results domain (parent code B), which focused on online user discussions related to public opinion about COVID-19 vaccine clinical trials, we were able to identify topics about the length (B-1-a, B-1-b), eligibility (B-2-[a-d]), the mechanism (B-3-[a-d]), stages (B-4-a), and the results (B-5-[a-h]) of existing COVID-19 vaccine clinical trials. Among all of these six sub-topics, the most dominant topic were discussions related to trial results (B-5[a-f], *n* = 798), including questions and answers of whether the results of specific COVID-19 vaccine clinical trials could be trusted and whether a vaccine approved based on those trial results could be considered safe (B-5-a, *n* = 247; B-5-d, *n* = 160); discussion about the results of trials taking place outside of the United States (B-5-b, *n* = 189); discussion about vaccine efficacy in different trials and efficacy for COVID-19 variants (B-5-c, *n* = 97; B-5-g, *n* = 55); discussion about purported safety events and long-term health effects (B-5-e, *n* = 39); and general public distrust of the results of trials based on claims about the vaccine manufacturer (B-5-h, n = 5). The second-largest sub-topic in this domain was related to mechanisms of current COVID-19 vaccine clinical trials (B-3-[a-d], *n* = 572). These conversations included discussions about: how efficacy of the vaccine is proven, are the participants being exposed to the COVID-19 virus during the trial (B-3-a, *n* = 163), what is the mRNA vaccine, and why is there a need for a placebo in a clinical trial (B-3-b, *n* = 151), how does the emergency use authorization impact the approval of a COVID-19 vaccine (B-3-c, *n* = 83), and what are the common processes and regulations of a clinical trial (B-3-d, *n* = 175). In this domain, we also observed topics that were related to the progress of different trials (B-4-a, *n* = 537); different opinions on the length of COVID-19 vaccine clinical trials (B-1-a, *n* = 12; B-1-b, a = 6); and conversations related to the eligibility to enroll in a trial (B-2-[a-d]), including whether there was sufficient participant representation (B-2-a, *n* = 59), health condition representation (B-2-b, *n* = 14), age representation (B-2-c, *n* = 42), and racial representation (B-2-d, *n* = 11).

**Fig. 2:**
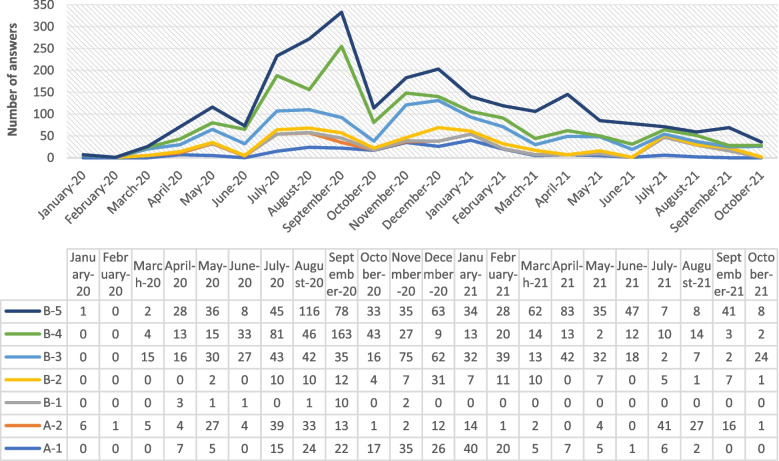
Timeline associated with topics

From a timeline perspective, the volume of the answers associated with COVID-19 vaccine clinical trials started increasing from the beginning of the data collection period until September 2020, representing the peak point of answers posted, and beginning in October 2020, the volume of answers started decreasing until the end of our data collection period. The most predominant topic detected earlier in the COVID-19 pandemic included discussion of participation in the COVID-19 vaccine clinical trial and a user debate regarding other countries’ vaccine development progress (A-2-a, B-5-b). Topics that emerged the latest in this study were associated with the eligibility of participating in trials, specifically, the discussion about different age representation in COVID-19 vaccine clinical trials (B-2-c) (see Fig. 2 for timeline associated with topics reviewed).

### Topic interaction analysis

Among all identified 508 Quora questions that were related to COVID-19 vaccine clinical trial topics, the number of answers for each question varied from a minimum of 1 answer to a max of 112 answers, averaging a 5.58 answer rate. The number of relevant answers for each question varied from a minimum of 1 answer to a maximum of 107 answers, averaging a 5.12 relevant answer rate. When analyzing the top 10 relevant answered questions (see Table 3), 3 of the questions (Question numbers 2,5,10) were related to clinical trial participation. These answered questions included users discussing their willingness to enroll their children or themselves into a COVID-19 vaccine clinical trial and the specific experiences of trial participation. The other 7 remaining questions were related to public opinion about COVID-19 vaccine clinical trials’ design/ process/ results (Question numbers 1,3,4,6,7,8,9), including asking how people think about other countries’ vaccine development progress, the safety of the approved vaccines, and if applicable regulations had been followed at trial completion.

**Table 3:** Top 10 answered questions

No.	Question	Answer number	Signal answer number	Topic theme code
1	How did Russia find a COVID-19 vaccine so fast? Could it have undergone all the proper trials in this short of time?	112	107	B
2	Would you enroll your kid for the COVID vaccine trial?	51	49	A
3	Why was AstraZeneca’s Covid-19 vaccine study put on hold?	46	46	B
4	Does the fact that the covid vaccines haven’t been tested on humans fully, mean that we are all unwittingly participating in a clinical trial that pharmaceutical companies are not having to pay their candidates?	43	32	B
5	If you’re a participant in a COVID-19 vaccine study, what can you tell us about your experience?	42	15	A
6	How is it possible to produce the vaccines for covid-19 without 10–15 years of proper clinical trials? Are the vaccines safe?	40	40	B
7	Is the Covid 19 vaccine on human trials successful?	39	36	B
8	Putin claims Russia has developed the world’s first COVID-19 vaccine, called “Sputnik.” Do you believe him? If you are Russian do you trust your government and medical institutions enough to be among the first to get this vaccine?	38	38	B
9	How can Covid vaccine makers guarantee long-term safety without prolonged trials?	37	32	B
10	Would you volunteer to test the COVID-19 vaccine?	33	33	A

**Fig. 3:**
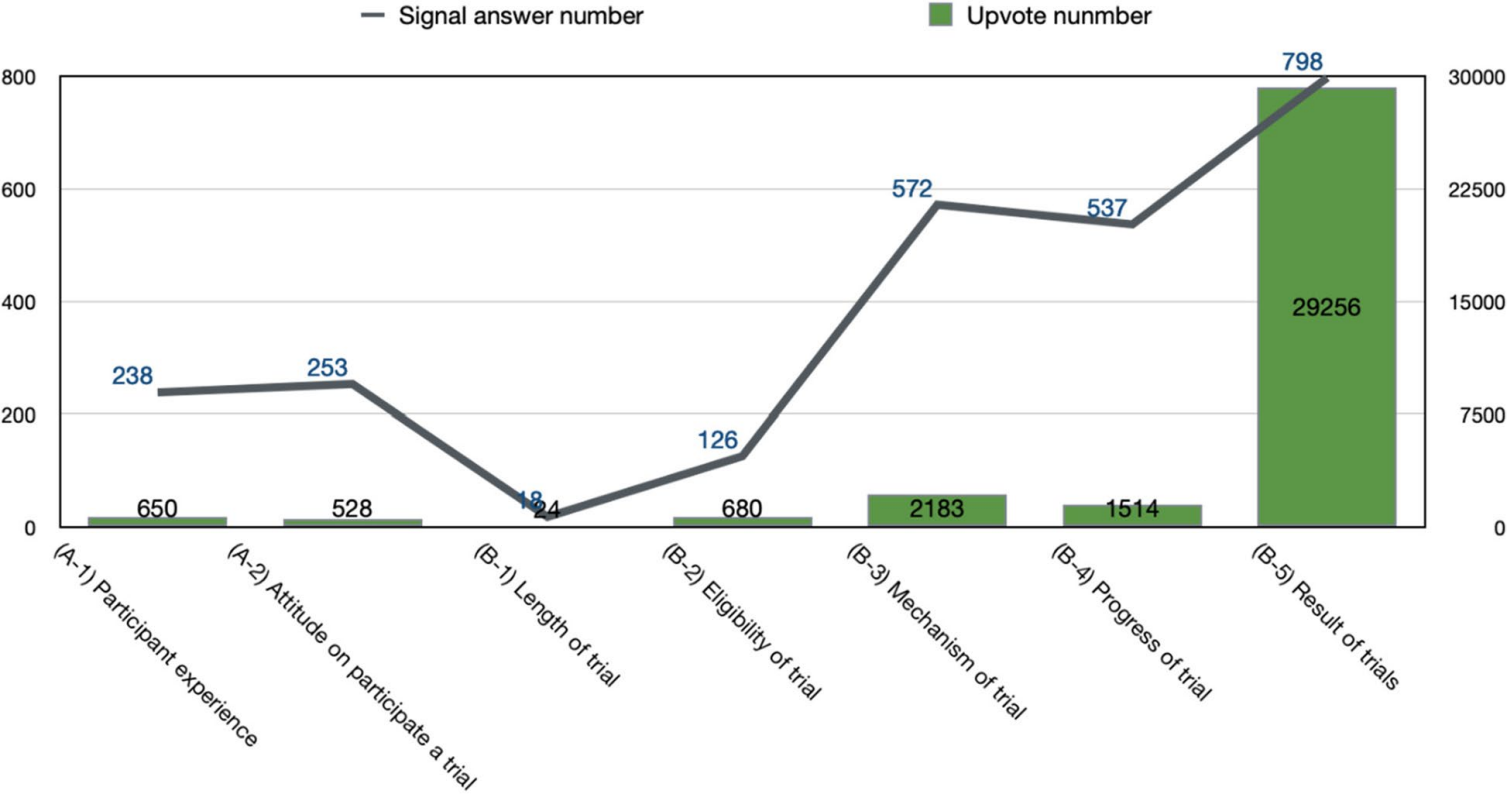
Relevant Answer and Upvote Number Breakdown into Sub-topics

According to all 2542 signal answers, we were able to find a total of 34,835 upvote interactions from 1400 relevant answers. The most accepted answer had 24,000 upvotes, though 142 answers did not have any upvoting. Based on the upvote number for the answers in each sub-topic, we observed discussions about the sub-topic related to results of COVID-19 clinical trials (B-5-[a-h]) had the most answers (*n* = 798) and upvoting interactions (*n* = 29,256, 83.98%) (See Fig. 3).

We also identified 4 questions and 12 answers with a total of 283 upvotes directly related to health equity topics, including questions discussing: (a) whether race is a social construct, and why is it important that COVID-19 vaccine trials involve a minimum number of racial or ethnic minority participants; (b) asking how many Blacks or African Americans have participated in COVID-19 vaccine trials; (c) discussion of why recruitment was difficult for certain racial or ethnic minority populations for COVID-19 vaccine trials; and (d) questions of why some COVID-19 vaccine trials had a lag time when enrolling racial and ethnic minority participants. The most upvoted answer (*n* = 140) explains why it is essential for a clinical trial to include participants from each racial or ethnic minority group.

### User metadata analysis

From 2542 relevant answers confirmed as related to COVID-19 vaccine clinical trial topics, 2033 (79.98%) answers had sufficient metadata in publicly available user profiles to identify the user’s reported country of residence, and 298 (11.72%) answer posts had self-identified racial or ethnic minority information. Among all 2033 answers with geographic location data, we observed that users reported they resided in 57 countries, and based on the distribution of these answers, the top five countries were the United States (1056/2033, 51.94%), England (282/2033, 13.87%), India (233/2033, 11.45%), Canada (113/2033, 6.54%), and Australia (89/2033, 4.38%). According to users’ racial and ethnic user profile information, we identified five answers posted from Black or African American users, 292 answers from Asian users, and one from a Hispanic or Latino user. Among all of the 298 answers from self-identified racial or ethnic minority group users, 30 of them (Black or African American = 5, Asian = 24, and Hispanic or Latino = 1) identified as current U.S. residents, with nine answers related to trial participation, including explaining to other users why they believed a trial was safe, discussing why as a Black or African American the user did not want to participate in any COVID-19 vaccine clinical trial, and sharing resources and knowledge about COVID-19 vaccine trials (A-1-[a,d,f],A-2-[a,d]). An additional 21 answers from racial and ethnic minority users were related to trial design/process and results, including expressing their trust of the COVID-19 vaccine, explaining to others their knowledge of the mRNA vaccine and the mechanism of the trial design, discussing why teenagers are not included in certain phases of a trial, as well as the discussion about the efficacy of certain vaccine trials. (B-2-b, B-3-[a-d], B-4-a, B-5-[a,c,e,g]).

## Discussion

Using our data mining and inductive content analysis approach, this study generated 508 questions and 2542 answers that were directly related to users interacting on a variety of topics relevant to the online discourse regarding COVID-19 vaccine clinical trials. Within the two major parent domains, there was a diversity of user conversation that encompassed 8-subcodes and 28 topics derived from these questions and answers that were explicitly focused on generating discussion, debate, and sharing knowledge, opinions, and lived experiences from participants about COVID-19 vaccine trials. Topics detected also elicited active user engagement, with an average of approximately 5 relevant answers for identified questions, providing evidencing that these online conversations not only have direct relevance to the topic of COVID-19 vaccine trials but also encourage user engagement. Specifically, we recorded 34,835 upvotes from users who interacted with relevant answers, though many came from one answer that had 24,000 upvotes specific to a question and answers regarding whether the COVID-19 vaccines should be deemed safe in the absence of 10 to 15 years of clinical research.

Importantly, this study detected topics in the parent domain of COVID-19 vaccine clinical trial participation from users who shared specific information about their own lived experiences while enrolling in a clinical trial, as well as users sharing opportunities to volunteer/enroll in a trial, and also users who conversely did not participate and explained why they would not enroll in a trial. Within this parent domain that made up 19.32% of our analyzed dataset, we detected specific conversations regarding concerns about the safety of vaccines and trials, sharing resources on how to participate in a vaccine trial, and also practical considerations about whether participating in a vaccine trial would preclude access to an approved vaccine during or after trial participation. These conversations directly relate to clinical trial recruitment and participation and are particularly relevant to the design of future clinical trial outreach strategies.

Overall, our parent domain of COVID-19 vaccine clinical trial design, process, and results discussions made up the majority of our dataset, comprising 80.68% of our question-and-answer online interactions, which also had the highest levels of user engagement through upvoting. Within this subcode generally comprising of trial knowledge sharing and opinions, topics varied significantly, including questions about the general safety of vaccine candidates, questioning the veracity of trials conducted in other countries, discussion about trust in vaccines based on characteristics of manufacturer and trial design, and concerns about long-term safety. Other dominant topics included conversations about trial eligibility (including discussion about adequate trial participation and racial and ethnic minority representation), progress (e.g., pauses in ongoing trials of the vaccine due to safety concerns), and mechanisms (i.e., questions and answers about trial design in relation to efficacy, vaccine development, and regulatory considerations) of trials. Most of these questions appear completely reasonable and warranted and some of our results align with other studies and public opinion polls that have identified concerns about confidence in the vaccine development and approval process, lack of understanding about trial design, concerns about safety and side effects, and concerns about the experimental nature of the clinical trial process, particularly in the context of abbreviated time periods or new vaccine platforms (e.g., mRNA) [12–14].

Additionally, within these questions and answers, a subset specifically addressed COVID-19 clinical trial and health equity topics, including very directed and focused conversations about the need for ensuring adequate racial and ethnic minority representation and enrollment in trials. Beyond questions and answers that were specific to health equity topics, approximately half of the users with location data were from the United States, and among users self-reporting their race or ethnicity, the majority were from users identifying as Asian, followed by a lower number of Black or African American, and a single Hispanic or Latino user. Though the availability and veracity of self-reported racial and ethnic profile data is uncertain, results demonstrate that though specific questions and answers may relate to clinical trial challenges specific to one racial group, users who participate in these online conversations may have a different composition. Results may also point to the need for more targeted online health communication and promotion efforts specifically designed to reach underserved communities through engagement with question-and-answer forums and other innovative digital outreach (e.g., virtual townhalls, etc.) [15].

### Limitations

This study has certain limitations. First, we only collected data from a single platform Quora and limited our study keywords to the English language. This likely biased study results to native English speakers, excluding minorities whom English is a second language or don’t speak English. Hence, the findings are not generalizable to all COVID-19 vaccine clinical trial Quora conversations occurring among online users. Our keywords related to COVID-19 vaccine clinical trial were also chosen based on our own manual searches on the platform but may have not been inclusive of all conversations related to the study aims, especially people using specific trial titles and their registration numbers. Additionally, we identified user's race and ethnicity status based only on user’s publicly available self-reported metadata. However, self-reported racial and ethnic status may be subject to misreporting by users, and we did not use other methods to validate user’s stated racial or ethnic minority representation. For purposes of user privacy and maintaining anonymity of data, we only present racial and ethnic minority results in the aggregate stratified by topics reviewed. All racial and ethnic self-reported data was only based on publicly available data from Quora answers reviewed. We also paraphrased example text of Quora answer results in order to ensure users cannot be readily re-identified.

## Conclusion

Our study is the first to our knowledge to examine user discussions on Quora related to the COVID-19 pandemic. Though prior published studies have examined Quora and other question-and-answer sites or platforms (e.g., Reddit, Yahoo! Answers, Wiki Answers, Stack Exchange, Zhihu) for topics as varied as food safety, multimorbid discussions, diabetes, mammography, radiology reports, and medical treatment and carbon reduction, ours is the only study to examine the confluence of COVID-19 pandemic, clinical trials for vaccines, and health equity topics in this group of specific question-and-answer interactions [16–21]. Major advantages of this infodemiology approach are the ability to generate insights into people’s attitudes, beliefs, behaviors, and experiences closer to real-time. These exploratory results should be further confirmed through more robust qualitative and quantitative research methods (e.g., focus groups and surveys) among a broader group of respondents, including those specifically from racial and ethnic minority groups. Additionally, though our approach relied on in-depth manual annotation of questions and answers, future studies should leverage unsupervised (e.g., topic modeling) and supervised machine learning approaches to classify online content more quickly and efficiently depending upon the availability and quality of training data. We hope that these results can help advance important considerations for improving the inclusivity and diversity of clinical trials, whether for COVID-19, vaccines, or other health conditions.

### Supplementary Information

**Additional file 1.** Standards for Reporting Qualitative Research (SRQR).

## Data Availability

Not applicable.

## Abbreviations

*COVID-19*: Coronavirus Disease of 2019
*mRNA*: Messenger Ribonucleic Acid
